# HIF‐1α regulates EMT *via* the Snail and β‐catenin pathways in paraquat poisoning‐induced early pulmonary fibrosis

**DOI:** 10.1111/jcmm.12769

**Published:** 2016-01-19

**Authors:** Yong Zhu, Jiuting Tan, Hui Xie, Jinfeng Wang, Xiaoxiao Meng, Ruilan Wang

**Affiliations:** ^1^ Department of Critical Care Medicine Shanghai First People's Hospital School of Medicine Shanghai Jiaotong University Shanghai China

**Keywords:** hypoxia‐inducible factor‐1α, epithelial‐mesenchymal transition, pulmonary fibrosis, paraquat

## Abstract

Paraquat (PQ) poisoning‐induced pulmonary fibrosis is one of the primary causes of death in patients with PQ poisoning. Hypoxia‐inducible factor‐1α (HIF‐1α) and epithelial‐mesenchymal transition (EMT) are involved in the progression of pulmonary fibrosis. Snail and β‐catenin are two other factors involved in promoting EMT. However, the relationship among HIF‐1α, Snail and β‐catenin in PQ poisoning‐induced pulmonary fibrosis is not clear. Our research aimed to determine whether the regulation of HIF‐1α in EMT occurs *via* the Snail and β‐catenin pathways in PQ poisoning‐induced pulmonary fibrosis. Sixty‐six Sprague–Dawley rats were randomly and evenly divided into a control group and a PQ group. The PQ group was treated with an intragastric infusion of a 20% PQ solution (50 mg/kg) for 2, 6, 12, 24, 48 and 72 hrs. A549 and RLE‐6TN cell lines were transfected with HIF‐1α siRNA for 48 hrs before being exposed to PQ. Western blotting, real‐time quantitative PCR, immunofluorescence, immunohistochemistry and other assays were used in our research. *In vivo*, the protein levels of HIF‐1α and α‐SMA were increased at 2 hrs and the level of ZO‐1 (Zonula Occluden‐1) was reduced at 12 hrs. *In vitro*, the transient transfection of HIF‐1α siRNA resulted in a decrease in the degree of EMT. The expression levels of Snail and β‐catenin were significantly reduced when HIF‐α was silenced. These data demonstrate that EMT may be involved in PQ poisoning‐induced pulmonary fibrosis and regulated by HIF‐1α *via* the Snail and β‐catenin pathways. Hypoxia‐inducible factor‐1α may be a therapeutic target for the treatment of PQ poisoning‐induced pulmonary fibrosis.

## Introduction

Over the last several decades, paraquat (PQ) has become a broadly used herbicide. However, PQ is highly toxic to humans and animals that come into direct contact with it, and in recent years, the incidence of PQ poisoning by suicide or accident has exhibited an increasing trend in Asia, especially in China 1, 2. The fatality rate of oral PQ poisoning is as high as 60–87.5%, and most of the survivors have pulmonary fibrosis, which has a poor prognosis 3, 4. Approximately 90% of ingested PQ accumulates in the lung within 6 hrs after PQ poisoning. Lung parenchyma cells become damaged, and the result is excessive repair of the lung tissues and finally the development of irreversible and extensive pulmonary fibrosis 5. However, the mechanism of PQ poisoning‐induced pulmonary fibrosis is still not clear, and there are no effective drugs or measures to treat these patients. In our previous study, we found collagen deposition in the lung tissues 2 hrs after PQ poisoning 6. This finding indicated that PQ poisoning‐induced pulmonary fibrosis emerges during the early stage of PQ poisoning. Therefore, a clear understanding of the molecular mechanism of PQ poisoning‐induced early pulmonary fibrosis is important for treating and reducing the mortality associated with PQ poisoning.

Hypoxia‐inducible factor‐1α (HIF‐1α) is a key mediator in cell metabolism, inflammation and tumorigenesis under hypoxic conditions 7, 8, 9. Recent studies have focused on the role of HIF‐1α in fibrosis diseases. Researches have shown that epithelial‐mesenchymal transition (EMT) takes part in the initiation and progression of fibrosis 10, 11, 12 and that HIF‐1α promotes the transformation of alveolar epithelial cells to fibroblasts 13. We found that HIF‐1α began to increase at 2 hrs after PQ poisoning 14. Thus, we speculate that HIF‐1α regulates the process of EMT in PQ poisoning‐induced early pulmonary fibrosis.

Snail and β‐catenin are two of the many EMT‐related regulators. Snail is a zinc finger transcription factor, which triggers the EMT by repressing expression of tight junction proteins, including E‐cadherin, claudin‐1, occludin and ZO‐1 15, 16, 17, 18. And β‐catenin, a main mediator of canonical Wnt signalling, plays a fundamental role in regulating cell proliferation and differentiation 19, 20. In normal condition, β‐catenin participates in the linking of E‐cadherin to the actin cytoskeleton. During EMT, β‐catenin dissociates from the E‐cadherin/β‐catenin cell membrane complexes, accumulates in the cytoplasm and translocates into the nucleus where it acts as a transcriptional coactivator through its binding with the members of the T cell factor/lymphoid enhancer factor (TCF/LEF) transcription factor family to promote transcription of genes that induce EMT 21, 22, 23. The regulation of HIF‐1α in EMT is partly modulated by Snail and β‐catenin in cancer 24, 25. In our previous research, we found a positive correlation among HIF‐1α, Snail and β‐catenin after PQ poisoning 26. However, the relationship among HIF‐1α, Snail and β‐catenin has not been extensively researched in pulmonary fibrosis. The aim of the present study was to confirm that HIF‐1α regulates EMT in PQ poisoning‐induced early pulmonary fibrosis and to further determine whether HIF‐1α modulates EMT *via* the Snail and β‐catenin pathways.

## Materials and methods

### Main drugs and reagents

Twenty per cent PQ stoste was obtained from Syngenta Crop Protection Ltd (Nantong, Jiangsu, China). The PQ stoste, paraformaldehyde and Triton X‐100 were obtained from Sigma‐Aldrich (St. Louis, MO, USA). DMEM and DMEM Nutrient Mixture F‐12 (Ham) (1:1) (DMEM/F‐12) were purchased from Gibco (Grand Island, NY, USA) and Hyclone (Logan City, UT, USA) respectively. Anti‐HIF‐1α, anti‐α‐Smooth Muscle Actin (SMA) and anti‐ZO‐1 primary antibodies were purchased from BioWorld (St. Louis Park, MN, USA), Abcam (Cambridge, MA, USA) and Santa Cruz Biotechnology Inc. (Santa Cruz, CA, USA) respectively. Anti‐Snail, anti‐β‐catenin, anti‐GAPDH and anti‐β‐actin primary antibodies were from Cell Signaling Technology (Boston, MA, USA). Their respective horseradish peroxidase (HRP)‐conjugated secondary antibodies were from Beyotime (Shanghai, China). Fluorescein isothiocyanate (FITC)‐conjugated goat anti‐rabbit IgG was purchased from Santa Cruz Biotechnology Inc.. Protein lysis buffer Radio Immunoprecipitation Assay (RIPA), Phenylmethanesulfonyl fluoride (PMSF), a Bicinchoninic Acid (BCA) protein concentration assay kit, an SDS‐PAGE gel preparation kit and 2‐(4‐Amidinophenyl)‐6‐indolecarbamidine (DAPI) were from Beyotime (Shanghai, China). PVDF membrane and highly sensitive Enhanced chemilumescent (ECL) agent were purchased from Bio‐Rad (Richmond, CA, USA) and Thermo Fisher Scientific (Waltham, MA, USA) respectively. An SABC immunohistochemistry kit was purchased from Boster (Wuhan, Hubei, China). TRIzol and Lipofectamine^™^ 2000 were purchased from Invitrogen (Grand Island, NY, USA). A SYBR^®^ Premix Ex Taq^™^ Kit and a Prime Script^™^ RT Master Mix Kit were purchased from TAKARA (Dalian, Liaoning, China).

### Animal models

Six‐ to eight‐week‐old male Sprague–Dawley (SD) rats were purchased from the Chinese Academy of Sciences experiment centre in Shanghai. All of the animal studies were approved by the Ethics Committee of Shanghai First People's Hospital. Sixty‐six healthy SD rats were randomly and evenly divided into a control group and a PQ group. Briefly, the PQ group was treated with an intragastric infusion of 20% PQ solution (50 mg/kg) and the control group received the same volume of saline. The PQ group was randomly divided into six subgroups according to the different times of examination (2, 6, 12, 24, 48 and 72 hrs), on average, after PQ treatment. Standard signs of PQ poisoning in rat models are as follows: loss of appetite, unresponsiveness, fluffy hair, rat‐tail cyanosis, dyspnoea, inability to support own weight and easy to catch 27, 28. According to the different time‐points, the rats were dissected after an intraperitoneal injection of sodium pentobarbital (50 mg/kg). The right lung lobe tissues were cryopreserved in liquid nitrogen, and the left lung lobe was kept in neutral formalin solution and embedded in paraffin for morphological examination.

### Cell culture

Cell lines of human lung adenocarcinoma epithelial cells A549 and rat alveolar type II cells RLE‐6TN were purchased from American Type Culture Collection (Rockville, MD, USA). In brief, A549 cells were cultured in DMEM with 10% foetal bovine serum (Gibco) and 1% antibiotics (100 U/ml penicillin, 0.1 mg/ml streptomycin). RLE‐6TN cells were cultured in DMEM/F‐12 with 10% foetal bovine serum and 1% antibiotics. Both of the cell lines were grown at 37°C in a 5% carbon dioxide incubator and were passaged following trypsinization.

### Real‐time quantitative PCR

Total RNA was extracted from tissues and cells with TRIzol reagent. The concentration of total RNA was detected using an ultraviolet spectrophotometer. Reverse transcription was carried out with a Prime Script^™^ RT Master Mix Kit according to the manufacturer's protocol. Real‐time quantitative PCR was executed with a SYBR^®^ Premix Ex Taq^™^ Kit in an Opticon Monitor 3 Sequence Detection System. The specific primers for β‐actin and HIF‐1α were generated by Sangon Biotech (Shanghai, China). The primer sequences of β‐actin and HIF‐1α are listed in Table 1. All of the samples were read in triplicate, and values were normalized to β‐actin.

**Table 1:** The primer sequences used in real‐time quantitative PCR

Species	Gene	Primer sequence (5′ → 3′; 3′ → 5′)
Homo sapiens	HIF‐1α	GTCTGAGGGGACAGGAGGAT; CTCCTCAGGTGGCTTGTCAG
	β‐actin	CTGGAACGGTGAAGGTGACA; AAGGGACTTCCTGTAACAATGCA
Rattus norvegicus	HIF‐1α	AAGTCTAGGGATGCAGCACG; AGATGGGAGCTCACGTTGTG
	β‐actin	AGGATGCAGAAGGAGATTACTGC; AAAACGCAGCTCAGTAACAGTGC

Primer sequences of β‐actin and HIF‐1α used in real‐time quantitative PCR. β‐actin serves as a loading control.

HIF‐1α: Hypoxia‐inducible factor‐1 alpha.

### Western blotting

Total protein from the rat lung tissue samples and the cells from each group was collected and extracted with RIPA. The protein concentrations were detected using a BCA protein assay kit. The total protein samples were separated on an 8% SDS‐PAGE and transferred onto a PVDF membrane, blocked with 5% non‐fat milk in Tris‐buffered saline with Tween 20 (TBST), and incubated overnight at 4°C with primary antibodies against HIF‐1α (1:500), α‐SMA (1:1000), ZO‐1 (1:500), Snail (1:500), β‐catenin (1:1000), GAPDH (1:500) and β‐actin (1:3000). The secondary antibodies, HRP‐conjugated goat anti‐rabbit IgG (1:2000) and goat antimouse IgG (1:2000), were incubated at room temperature. After washing in TBST, the immunoreactive bands were visualized with the ECL detection system according to the manufacturer's protocol.

### Immunofluorescence analysis

A549 and RLE‐6TN cells were grown in confocal dishes and treated with PQ. After 24 hrs, the cells were washed twice with PBS, fixed with 4.0% paraformaldehyde in PBS for 10 min., and permeabilized with 0.5% Triton X‐100 in PBS for 10 min. at room temperature. The cells were blocked in 5% bovine serum albumin for 60 min. at room temperature and then incubated with HIF‐1α antibody (1:50) at 4°C overnight. The cells were washed three times with TBST and incubated with FITC‐conjugated goat anti‐rabbit IgG (1:100) for 90 min. at room temperature. Nuclei were stained with DAPI for 3 min. after rinsing the cells with TBST. Finally, the fluorescence signals were observed under a laser confocal scanning microscope (Leica TCS SP8; Leica, Wetzlar, Germany).

### Immunohistochemistry

Immunostaining of HIF‐1α was performed on 5‐μm‐thick sections of the rat lung tissues. Subsequently, the tissue slides were incubated for 2 hrs at 67°C and de‐paraffinized. The endogenous peroxidase activity was blocked for 10 min. with a 3% hydrogen peroxide‐methanol solution at room temperature. Antigen retrieval was performed with a microwave twice, each time for 8 min. After blocking with 5% BSA for 20 min. at room temperature, the samples were incubated with HIF‐1α antibody (1:100) at 4°C overnight. The slides were rinsed in PBS and incubated with the secondary antibody (1:2000) for 30 min. at 37°C. Then, the slides were incubated with SABC reagent at 37°C for 20 min. and washed with PBS 4 times for 5 min. each time. Next, the slides were incubated with DAB and haematoxylin stain for 40 sec., differentiated in 1% hydrochloric acid alcohol, and then rinsed in tap water for 10 min. Subsequently, the slides were dehydrated in an alcohol gradient, made transparent with xylene and mounted with neutral gum. Finally, we observed and photographed the results under a microscope.

### Transient transfection

Hypoxia‐inducible factor‐1α siRNA and scrambled control sequences were obtained from GenePharma (Shanghai, China). Transient transfection of cells with HIF‐1α siRNA and scrambled control sequences was performed with Lipofectamine 2000 in accordance with the manufacturer's protocol but with slight modifications. After 48 hrs, the cells were harvested and real‐time PCR and western blotting assays were performed.

### Cell viability assays

The effects of PQ on cell viability were determined with a Cell Counting Kit‐8 (CCK‐8) from Dojindo (Kumamoto, Kyushu, Japan). A549 cells (5 × 10^3^/well) and RLE‐6TN cells (8 × 10^3^/well) were seeded in 96‐well plates 24 hrs prior to PQ treatment. The concentration gradients of PQ to A549 cells were 0, 200, 400, 600, 800, 1000 and 1200 μmol/l, and those of RLE‐6TN cells were 0, 40, 80, 120, 160 and 200 μmol/l. The same volume of CCK‐8 solution was added to each well after 24 hrs of PQ treatment and incubated for 3 hrs at 37°C. The absorption value of each well was measured with a Multimode Reader. Independent assays were performed three times in triplicate.

### Statistical analysis

All of the data were statistically analysed using SPSS (version 16.0; Chicago, IL, USA). Three independent experiments were performed. Measurement data are expressed as the mean ± S.D. A *t*‐test was used for comparisons between two groups. Statistical significance was set at *P* < 0.05.

## Results

### EMT is involved in PQ poisoning‐induced pulmonary fibrosis

**Figure 1:**
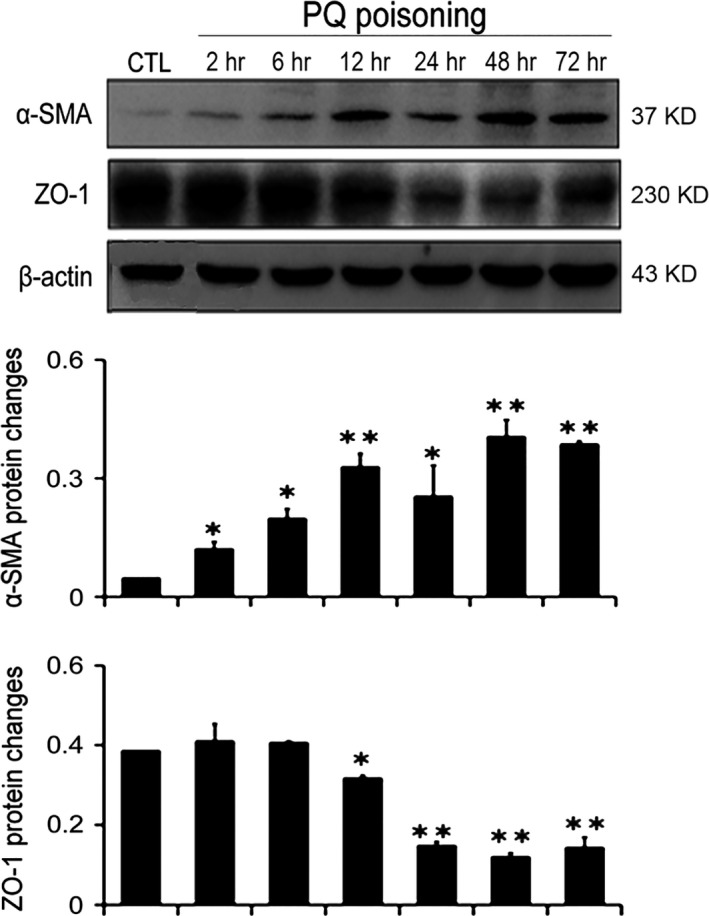
The expression of EMT‐related biomarkers in PQ‐poisoned rat lung tissues. The levels of α‐SMA and ZO‐1 in the rat lung tissues were detected by Western blotting. Bar graphs show the fold changes of α‐SMA and ZO‐1 proteins related to β‐actin. β‐actin served as a loading control. The data are shown as the mean ± S.D. *Significantly (*P* < 0.05) different from CTL; **Significantly (*P* < 0.01) different from CTL. CTL denotes the control group.

To demonstrate that EMT is associated with the development of PQ poisoning‐induced pulmonary fibrosis, we detected the expression of α‐SMA and ZO‐1, which are markers of EMT, by Western blotting 29. α‐SMA, a mesenchymal cell marker protein, was increased at 2 hrs, and ZO‐1, an epithelial cell marker protein, was significantly decreased at 24 hrs (Fig. 1). These results confirm that EMT is involved in PQ poisoning‐induced early pulmonary fibrosis.

### HIF‐1α expression increased in the PQ‐poisoned rat lung tissues and alveolar epithelial cells

**Figure 2:**
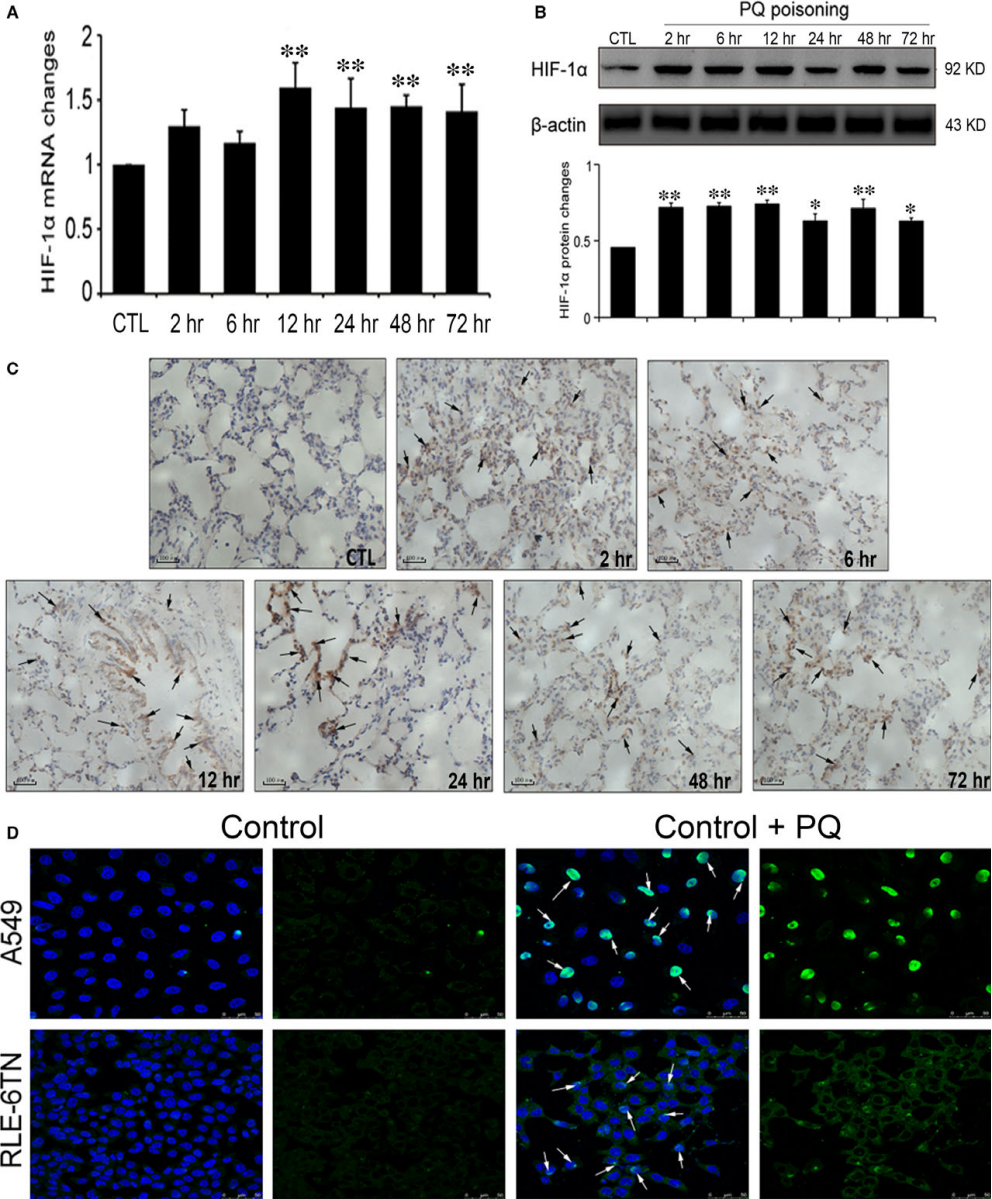
The expression of HIF‐1α in the PQ‐poisoned rat lung tissues and alveolar epithelial cells. (**A**) Rat lung tissues were examined at different time‐points after PQ treatment. The expression level of HIF‐1α mRNA was examined by real‐time quantitative PCR. β‐actin served as a loading control. (**B**) HIF‐1α and β‐actin in the rat lung tissues were detected by Western blotting. Bar graphs show the fold change of HIF‐1α protein. (**C**) The level of HIF‐1α protein in the rat lung tissues was detected by immunohistochemistry, scale bars = 100 μm. Arrows indicate HIF‐1α positive staining. (**D**) The expression of HIF‐1α protein in the A549 and the RLE‐6TN cell lines was detected by immunofluorescence, scale bars = 50 μm. Arrows indicate HIF‐1α positive staining. The data are shown as the mean ± S.D. *Significantly (*P* < 0.05) different from CTL; **Significantly (*P* < 0.01) different from CTL. CTL denotes the control group.

To investigate the role of HIF‐1α in PQ poisoning‐induced early pulmonary fibrosis, we detected the level of HIF‐1α in PQ‐poisoned rat lung tissues and alveolar epithelial cells using real‐time quantitative PCR, Western blotting, immunofluorescence and immunohistochemistry. The level of HIF‐1α mRNA in the PQ‐poisoned rat lung tissues, which was detected by real‐time quantitative PCR, was significantly increased after 12 hrs of PQ poisoning and was maintained at increased levels up to 72 hrs (Fig. 2A). The results of Western blotting indicated that HIF‐1α protein expression was markedly increased at 2 hrs compared with the control group (Fig. 2B). The same result was found by immunohistochemistry, which revealed several brown HIF‐1α protein particles in the alveolar wall and the bronchial wall at 2 hrs. Disorder of the alveolar structure was widely present; more cells had a patchy distribution, and the brown particles were widely distributed after exposure to PQ for 72 hrs (Fig. 2C). In the both the A549 and RLE‐6TN cell lines, the results of immunofluorescence showed that the expression of HIF‐1α was significantly increased in the nucleus after treatment with PQ for 24 hrs (Fig. 2D). These results indicated that HIF‐1α participates in the initiation and development of PQ poisoning‐induced early pulmonary fibrosis.

### The process of PQ poisoning‐induced EMT may be regulated by HIF‐1α

**Figure 3:**
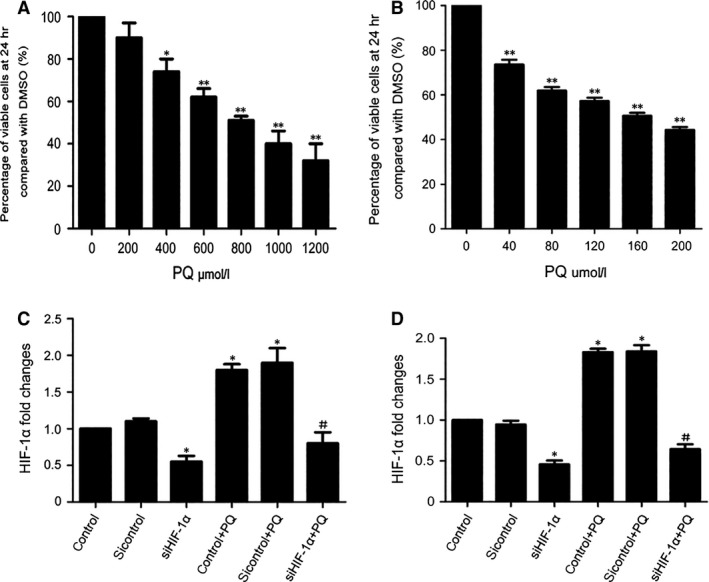
Cells viability after PQ treatment and the level of HIF‐1α mRNA after transiently transfecting HIF‐1α siRNA in both cell lines. (**A** and **B**) The A549 and RLE‐6TN cell lines were treated with different concentrations of PQ for 24 hrs. Cell viability was detected with a Cell Counting Kit‐8. (**C** and **D**) Both cell lines were transfected with HIF‐1α siRNAs for 48 hrs and treated with PQ for 24 hrs. The level of HIF‐1α mRNA was detected by real‐time quantitative PCR. β‐actin was the loading control. The data are shown as the mean ± S.D. *Significantly (*P* < 0.05) different from control or 0 group; **Significantly (*P* < 0.01) different from 0 group; ^#^Significantly (*P* < 0.05) different from control + PQ.

According to many researchers, HIF‐1α might promote EMT *via* a number of pathways 24, 30. In tumours, HIF‐1α promoted the expression of transforming growth factor (TGF)‐β, LOX, Snail, TWIST‐1, TCF‐3, ZEB1 (Zinc finger E‐box‐binding homeobox 1) and ZEB2, which then activated EMT and the metastasis of tumour cells 31, 32, 33, 34. In renal fibrosis, Bim1 mediated the regulatory role of HIF‐1α in EMT in tubular epithelial cells 30. The data above showed that HIF‐1α increased prior to EMT. Thus, we attempted to demonstrate that HIF‐1α regulated EMT in PQ poisoning‐induced early pulmonary fibrosis. First, we determined the median lethal dose of PQ to both the A549 and the RLE‐6TN cell lines using the CCK‐8. The results showed that the concentrations of PQ in treated cells at 24 hrs was 800 μmol/l for the A549 cells (Fig. 3A) and 160 μmol/l for the RLE‐6TN cells (Fig. 3B). Next, HIF‐1α siRNAs were transiently transfected into both cell lines, which significantly reduced HIF‐1α mRNA (Fig. 3C and D) levels in the DMSO group and the PQ group.

**Figure 4:**
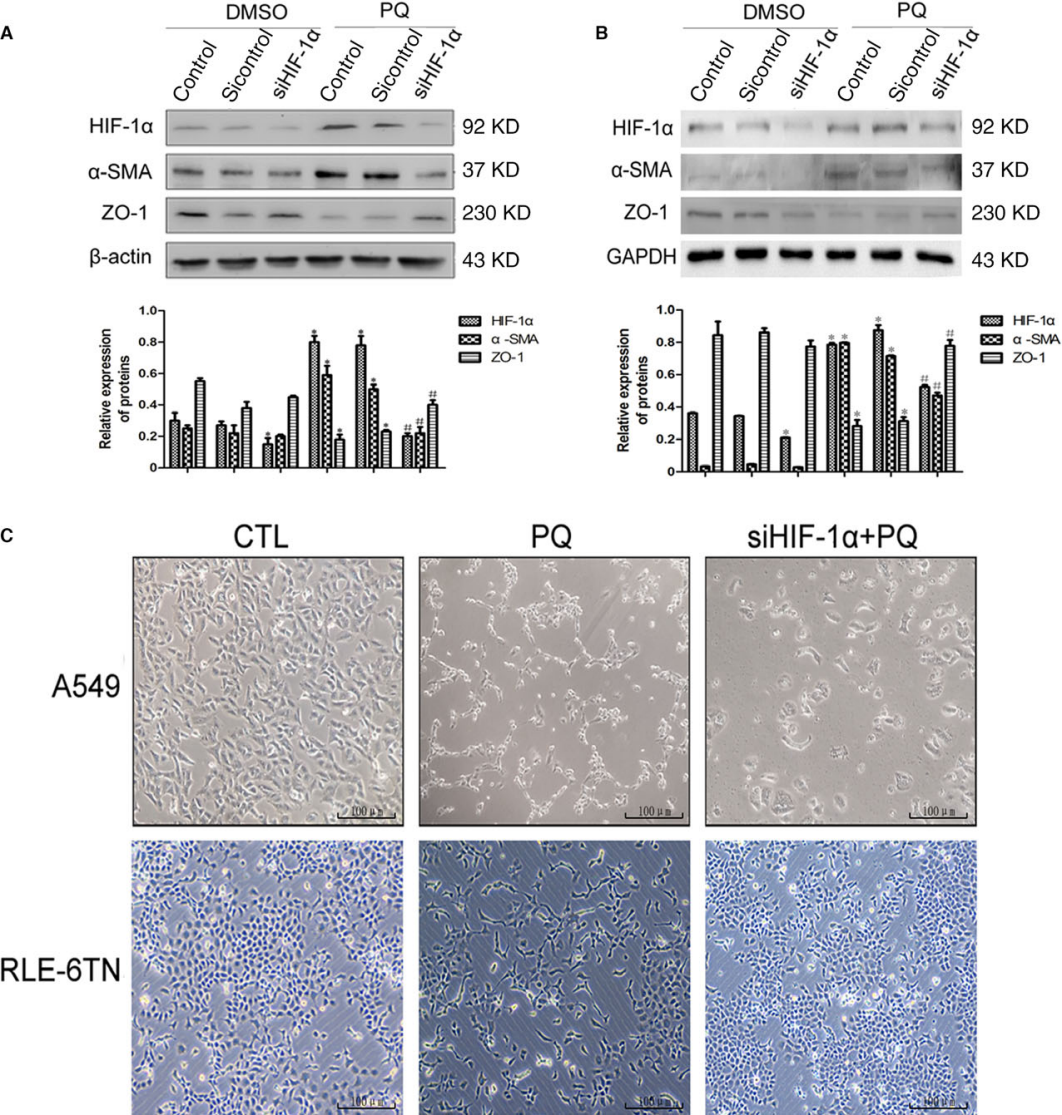
Changes in EMT after silencing HIF‐1α *in vitro*. (**A** and **B**) The expression levels of HIF‐1α, α‐SMA and ZO‐1 proteins were detected by Western blotting in both cell lines after silencing HIF‐1α. β‐actin and GAPDH served as loading controls. (**C**) The morphological changes in the A549 and RLE‐6TN cell lines under the phase contrast microscope after silencing HIF‐1α, scale bars = 100 μm. The data are shown as the mean ± S.D. *Significantly (*P* < 0.05) different from control; #Significantly (*P* < 0.05) different from control + PQ.

In the A549 (Fig. 4A) and the RLE‐6TN cell lines (Fig. 4B), the results of Western blotting showed that the expression of α‐SMA was increased and the expression of ZO‐1 was reduced in the PQ group compared with the DMSO group. In contrast, when HIF‐1α expression was silenced, the level of α‐SMA was reduced and the level of ZO‐1 expression was increased in the PQ group. We also observed the morphological changes in the cells under a phase contrast microscope, The cells changed from polygons or a cobblestone morphology to fusiform in the PQ group, but the degree of fusiformity was reduced after the silencing of HIF‐1α expression (Fig. 4C). These *in vitro* results further suggested that EMT acts as a mechanism in PQ poisoning‐induced early pulmonary fibrosis and that HIF‐1α is an important modulator of this process.

### HIF‐1α may regulate EMT *via* the Snail and β‐catenin pathways

**Figure 5:**
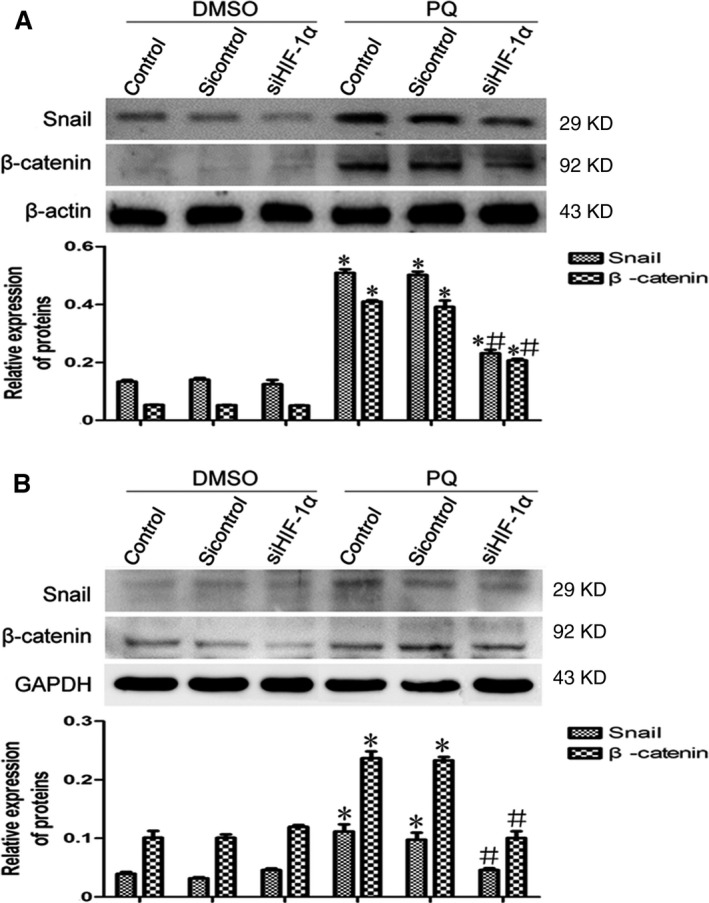
The expression levels of Snail and β‐catenin after silencing HIF‐1α *in vivo*. (**A** and **B**) The expression levels of Snail and β‐catenin in the A549 and RLE‐6TN cell lines were assessed by Western blotting after silencing HIF‐1α. β‐actin and GAPDH served as the loading controls. The data are shown as the mean ± S.D. *Significantly (*P* < 0.05) different from control; ^#^Significantly (*P* < 0.05) different from control + PQ.

**Figure 6:**
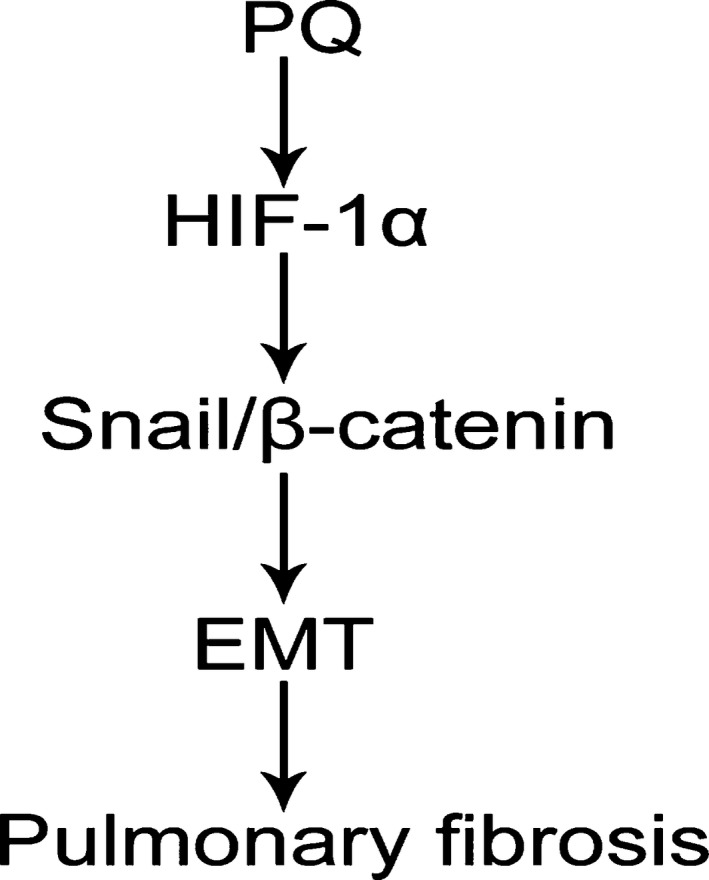
Potential signalling pathways of HIF‐1α participating in PQ poisoning‐induced pulmonary fibrosis. PQ might promote the development of pulmonary fibrosis through HIF‐1α signalling, which may regulate the Snail and β‐catenin pathways to induce EMT.

Transforming growth factor‐β1 is a classical EMT‐promoting cytokine, and many studies have shown the relationship between HIF‐1α and TGF‐β1 in the process of EMT 13, 35, 36. We also found that TGF‐β1 has a positive correlation with HIF‐1α as well as Snail and β‐catenin in PQ poisoning‐induced pulmonary fibrosis, the latter of which are the two other EMT‐promoting cytokines in addition to TGF‐β1 26. To further investigate the interaction among HIF‐1α, Snail and β‐catenin, we measured the expression changes of Snail and β‐catenin by Western blotting after silencing HIF‐1α expression *in vitro*. As shown in the Western blotting results, we found that the expression of Snail and β‐catenin was markedly reduced when HIF‐1α was silenced in the A549 (Fig. 5A) and the RLE‐6TN cell lines (Fig. 5B). These data indicated that the role of HIF‐1α in the EMT process might occur *via* the Snail and β‐catenin pathways (Fig. 6).

## Discussion

Although the mechanism of PQ poisoning primarily involves the formation of oxidative stress over time, the major problem in treating PQ poisoning is the development of PQ poisoning‐induced early pulmonary fibrosis 5. The molecular mechanism of PQ poisoning‐induced early pulmonary fibrosis remains unclear, and there is no effective method to prevent its occurrence and development. Our study showed that HIF‐1α might modulate EMT *via* the Snail and β‐catenin pathways in PQ poisoning‐induced early pulmonary fibrosis. Therefore, HIF‐1α may be a therapeutic target for preventing the occurrence and progression of PQ poisoning‐induced pulmonary fibrosis in the early stage.

A recent study demonstrated that the main features of fibrosis are the activation of fibroblast proliferation and extracellular matrix deposition 37. The activated fibroblasts usually have three sources: the proliferation of fibroblasts around the injury site, the EMT, and the migration of proliferating and differentiating fibrocytes from the circulation to the lungs 38. The excessive activation of fibroblasts and the collagen deposition in the extracellular matrix coincide with the occurrence of EMT 39. It has been demonstrated that alveolar epithelial cells, especially type II alveolar epithelial cells, acquire the phenotype of fibroblasts through EMT to promote the development of pulmonary fibrosis 40, 41. A549 cells retain the features of type II alveolar epithelial cells although they are a type of cancer cells 42, and this cell line is widely used to study the mechanism of pulmonary fibrosis 43, 44, 45. In alveolar epithelial cells, decreased ZO‐1 and increased α‐SMA are reliable indicators of the occurrence of EMT 29. Here, our research showed that alveolar epithelial cells acquired a mesenchymal cell phenotype at the early stage of PQ poisoning both *in vivo* and *in vitro*. We confirmed that EMT takes part in the progression of PQ poisoning‐induced early pulmonary fibrosis. Thus, a clear understanding of the EMT mechanism in the lung epithelial cells after PQ poisoning and blocking this mechanism early might be an important component of the treatment of PQ poisoning‐induced pulmonary fibrosis.

In addition to TGF‐β1, a prototypical cytokine for the induction of EMT, HIF‐1α promotes the transformation of alveolar epithelial cells into mesenchymal cells 13, 46. Recent studies have shown that HIF‐1α, as a profibrotic transcription factor, is involved in a variety of organs during the EMT process 47, 48. In our study, HIF‐1α protein expression increased in the early stage of PQ poisoning (2 hrs) sooner than HIF‐1α mRNA levels (12 hrs), which suggests that HIF‐1α is activated first at the translational or posttranslational level. Hypoxia‐inducible factor‐1α protein degradation was inhibited and its gene expression increased 12 hrs later. We found that the degree of PQ‐induced EMT in lung epithelial cells was alleviated after silencing HIF‐1α expression. This finding indicated that HIF‐1α may regulate EMT in PQ poisoning‐induced pulmonary fibrosis.

Further study of the regulatory mechanism of HIF‐1α in EMT revealed that Snail and β‐catenin, two other EMT regulatory factors, were markedly reduced after silencing HIF‐1α expression. Reports have shown that Snail plays an important role in the initiation of EMT 49, 50. Some studies have demonstrated that β‐catenin may be involved in the formation of pulmonary fibrosis by driving A549 cells to fibroblasts 51, 52. And in tumours, β‐catenin which accumulates in the nucleus co‐activates with TCF/LEF, which controls transcription of genes that induce EMT, such as Snail, TWIST and ZEB 29, 53, 54. In renal fibrosis, some researches indicated that both Snail and β‐catenin participate in the development of EMT and the expression of Snail was regulated by β‐catenin 55, 56. However, both the expression of Snail and β‐catenin were regulated by HIF‐1α in this study. And researches have also shown that HIF‐1α could directly regulate the expression of Snail in the development of EMT 57, 58. In our previous research, we found a positive relationship among Snail, β‐catenin and HIF‐1α after PQ poisoning 26. Thus, it may exist a complex relationship between Snail and β‐catenin in PQ poisoning‐induced EMT. In this study, we have not studied the interaction between Snail and β‐catenin in PQ poisoning‐induced pulmonary fibrosis, which needs to be further investigated. These data suggested that HIF‐1α may promote PQ poisoning‐induced early pulmonary fibrosis by regulating EMT *via* the Snail and β‐catenin pathways.

In conclusion, our study further elucidates the pathogenesis of PQ poisoning‐induced early pulmonary fibrosis. We found that HIF‐1α expression increases and that alveolar epithelial cells acquire a mesenchymal cell phenotype in PQ poisoning‐induced early pulmonary fibrosis. In addition, we confirmed that HIF‐1α takes part in the PQ‐induced EMT process and that the mechanism of HIF‐1α‐regulated EMT may occur *via* the Snail and β‐catenin pathways. Thus, HIF‐1α might be a target for the treatment of PQ poisoning‐induced early pulmonary fibrosis.

## Conflicts of interest

The authors confirm that there are no conflicts of interest.
